# Application of Pulsed Electric Field as a Pre-Treatment for Subcritical Water Extraction of Quercetin from Onion Skin

**DOI:** 10.3390/foods11081069

**Published:** 2022-04-07

**Authors:** Han-Sol Kim, Min-Jung Ko, Chan-Ho Park, Myong-Soo Chung

**Affiliations:** 1 Department of Food Science and Engineering, Ewha Womans University, Seoul 03760, Korea; khsooool@naver.com; 2 Department of Food Science and Biotechnology, Global K-Food Research Center, Hankyong National University, Anseong-si 17579, Korea; mjko@hknu.ac.kr; 3 R&D Center, Ottogi Corporation, Anyang-si 14060, Korea; chpark@ottogi.co.kr

**Keywords:** onion skin, by-product, quercetin, subcritical water extraction, pulsed electric filed

## Abstract

Pulsed electric field (PEF) treatment promotes the electroporation of biological membranes, and if included as a pre-treatment, could improve the extraction of certain constituents therefrom. The aim of this study was to determine the optimal extraction conditions for extracting the flavonoid quercetin from dried onion skin and to establish whether the yield could be enhanced by combining PEF pre-treatment with an eco-friendly extraction method that uses subcritical water extraction (SWE). Samples of onion skin were treated with PEF under conditions of varying electric field strength (0.5–2.5 kV/cm) and duration (5–120 s). SWE was then performed with an extraction time of 15 min and at temperatures ranging from 105 °C to 185 °C. Among the conditions tested, the yield of total quercetin was the highest after pretreatment with PEF at 2.5 kV/cm for 15 s, followed by SWE at 145 °C for 15 min (yield 19.25 ± 0.77 mg/g dried onion skin, mean ± standard deviation). Pretreatment with PEF improved the yield of total quercetin extracted by 33.22% compared with the PEF-untreated samples. These findings demonstrate that pretreatment of onion skin with PEF has the potential to improve flavonoid extraction.

## 1. Introduction

Onions (*Allium cepa* L.) are vegetables that are widely consumed in many countries. They contain flavonoids such as quercetin and quercetin glucosides, which have been shown to confer health benefits, including antioxidant, and anti-inflammatory activities, in humans [1,2,3]. Onions are included in human diets that are designed to promote preventive healthcare through the intake of fruits and vegetables. Onions have been cultivated throughout the world for a long time, and their production and the areas of cultivation have continued to increase. However, the amount of onion waste is enormous; for example, the European Union generates more than 500,000 tons of onion waste annually [4]. Given that about 10% of every onion is discarded or used as compost, it may be economically useful to determine the physiological usefulness and feasibility of using discarded onion skin as an edible product. The food industry currently produces a significant number of phenol-rich by-products, which are a natural source of antioxidants and are used as ingredients for food stuffs [5]. Processing waste such as onion skins may provide the dual benefits of solving an environmental problem resulting from the disposal of onion waste while simultaneously acquiring stabilized onion by-products to use as natural antioxidant food ingredients. The most efficient method of extraction of phenol compounds from onion skin should thus be sought, including methods for enhancing the yield. Plant-based flavonoids are attracting considerable attention due to the increased antipathy for synthetic products and increasing preferences for natural compounds [6]. As a result, there has been an increasing need for practical methods of obtaining these bioactive compounds from various plant sources [7]. In order to utilize the bioactive compounds contained in plants, these materials must be isolated from the raw materials. Methods of flavanol extraction from onion have been investigated in many studies [8,9,10]. Traditional extraction methods are generally time-consuming and involve the use of organic solvents such as methanol and ethanol, which are toxic to humans.

A method of bioactive compounds extraction that uses subcritical water has potential as an alternative to traditional methods that use organic solvents, providing the advantages of being both environmentally safe and nontoxic to humans. Subcritical water extraction (SWE) is a green technique due to using only purified water for solvents. Subcritical water is a liquid state held by pressure at a temperature between the atmospheric boiling point (100 °C) and the critical temperature (374 °C) of water. At a high temperature, a high pressure can be used to maintain the water in a liquid state [11]. As the water heats to temperatures well above 100 °C, its dielectric constant decreases so that it mimics the solubility of organic solvents such as methanol and ethanol for low-polarity compounds [12]. This means that subcritical water could be an alternative solvent for extracting less-polar compounds. This method is also a rapid, low-cost, non-toxic solvent, and has a high extraction efficiency. Our previous studies have indicated that the extraction yield of quercetin from onion skin in the subcritical water condition was over eight-, six-, and four-fold higher than those obtained by conventional extraction methods using ethanol, methanol, and water at boiling point [13].

Pulsed electric field (PEF) technology is a nonthermal technique in which a very short voltage pulse with a high electric field strength (*E*) is applied to a biological material located between two electrodes, causing physical destruction of cell membranes [14]. PEF causes electroporation of the cell membranes, promoting the leakage of intercellular compounds. At low values of *E* (0.2–1 kV/cm), plant tissue can be destroyed without a marked increase in temperature [15]. PEF is used as a pretreatment in the preparation of fruit juices, increasing the yield and enhancing the extraction of antioxidants, colorants, or flavors from cells, and improving the drying and freezing process. PEF pretreatment has been shown to increase the extraction efficiency of hesperidin and naringin from orange peel, as well as that of the total phenolic contents from grapes [16,17]. These findings suggest that PEF pretreatment could help to extract useful components from vegetables. Therefore, PEF-assisted extraction could be an attractive industrial food processing technology for extracting bioactive compounds. Although many studies related to PEF as a pretreatment extraction have been done, there are no reports about combining the assisted PEF with SWE technology for extracting quercetin from onion skin.

In the present study, we investigated the effectiveness of PEF as a pre-treatment, and SWE for extracting the flavanols quercetin and quercetin glycoside from onion skin. The aims of this study were three-fold: (i) to determine whether the efficiency of SWE of quercetin and quercetin glycosides from onion peel could be improved without the use of organic solvents such as ethanol and methanol by increasing cell permeation by PEF pretreatment; (ii) to explore whether the extraction efficiency could be improved by combining PEF and SWE, and during that process determine the optimal PEF and SWE conditions for extraction; and (iii) to determine the effect of combined PEF pretreatment and SWE on the concentration of flavanols extracted from onion skin.

## 2. Materials and Methods

### 2.1. Sample Preparation

Samples of yellow onion skin (*Allium cepa* L.) were purchased from Muan (Jeollanam-do, Korea), with only those with outer skins being used. The moisture content of the onion-skin samples was 9–12%. The onion skins were cut into pieces smaller than 10 mm before PEF treatment. All of the samples were stored in a refrigerator maintained at 4 °C until they were required for use, and were soaked in 270 mL of tap water prior to the application of PEF.

### 2.2. Chemicals and Reagents

High-performance liquid chromatography (HPLC)-grade solvents were used. Methanol and water were purchased from J. T. Baker (Phillipsburg, NJ, USA), and formic acid was obtained from Sigma-Aldrich (St. Louis, MO, USA). Onion-skin extracts were identified and quantified using standard compounds, such as quercetin aglycone and quercetin glycoside (Extrasynthese, Genay, France).

### 2.3. PEF Pre-Treatment of Onion Skins

**Figure 1:**
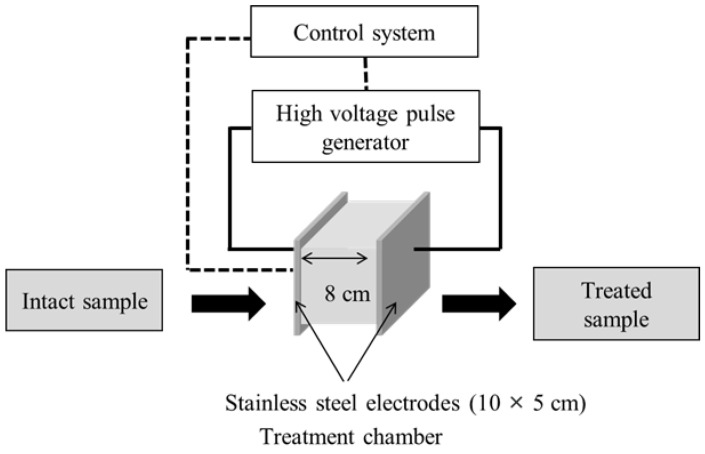
Schematic diagram of the PEF device (HVP-5, DIL).

PEF pre-treatment was performed using a 5-kW pulse generator (HVP-5, DIL, Quakenbrueck, Germany) (Figure 1). A batch-type treatment chamber (10 cm × 5 cm × 8 cm) was used that comprised two parallel stainless-steel electrodes (10 × 5 cm) separated by 8.0 cm. PEF was applied to 15-g samples of onion skin that had been soaked in tap water, at a pulse frequency and width of 50 Hz and 25 μs, respectively. Experiments to test the parameters of PEF that improved the onion-skin membrane permeability were conducted at *E* values of 0.5, 1.0, 2.0, and 2.5 kV/cm. The effect of the PEF *E* value on extraction efficiency was investigated at a fixed PEF treatment time of 60 s. The optimal extraction conditions were evaluated by treating onion-skin samples at *E* = 2.5 kV/cm for 5, 15, 30, 60, 90, and 120 s, followed by SWE at 105 °C, 125 °C, 145 °C, 165 °C, and 185 °C for 15 min. The SWE-harvested extracts were stored at 4 °C until required for analysis.

### 2.4. Determination of the Cell Disintegration Index

Membrane conductivity was measured with a conductivity meter (CP-50N, ISTEK, Seoul, Korea) and a 5731302A IH conductivity probe to determine the extent of the cellular structural damage caused by the extraction process. Each experiment was repeated three times with water-soaked onion skin waste. The degree of cell damage was quantified by calculating the concentration of charged particles using the electrical conductivity disintegration index, *Z* [18], as follows:

*Z* = (σ − σ_0_)/(σ_d_ − σ_0_) (1)

where σ is the electrical conductivity of the sample measured with a conductivity meter (μS/cm), and σ_0_ and σ_d_ represent the conductivities of the undamaged and completely destroyed samples, respectively, and σ_d_ was determined by repeatedly freezing and thawing the samples. According to this definition, *Z* = 0 indicates a complete onion-skin sample and *Z* = 1 indicates a totally destroyed sample.

### 2.5. Extraction of Flavonols Using SWE

**Figure 2:**
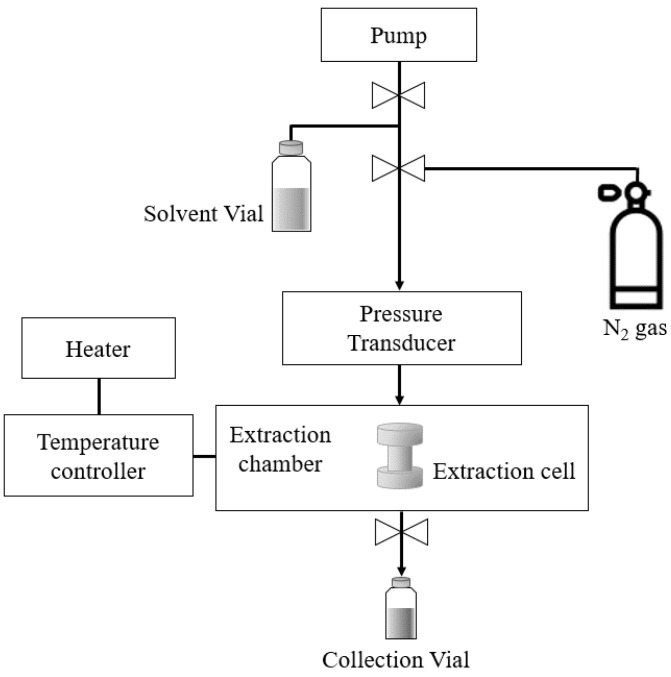
Schematic diagram of SWE (ASE 350, Dionex).

SWE was performed using an accelerated solvent extractor (ASE 350, Dionex, Sunnyvale, CA, USA) (Figure 2). Ultrapure water supplied by a Milli-Q device (MR-RO800, Mirae ST, Anyang, Korea) was used as an extraction solvent. Wet onion-skin samples (4 g) pretreated with PEF were used for extraction. Immediately, each sample was loaded into a 22-mL extraction cell (23 mm inner diameter × 50 mm long; Dionex) containing a single sheet of filter paper on the bottom. For SWE, the extraction cell containing the solvent was heated under a pressure of about 100 bar using nitrogen gas. The onion-skin extracts were collected into vials (25–35 mL). SWE was conducted at several different temperatures (105–185 °C) with an extraction time of 15 min.

### 2.6. HPLC Analysis

The onion-skin extract was analyzed after it had cooled to room temperature. The extract volume was made up to 100 mL with methanol, and passed through a 0.45-μm polyvinylidene fluoride filter (Whatman, Maidstone, Kent, UK). A 0.1-mL sample of filtered extract was mixed with 0.9 mL of a solution of methanol/formic acid/water (50:5:45, *v*/*v*/*v*) stabilized with 2 g/L of 98% tert-butylhydroquinone (Toronto Research Chemicals, Toronto, Ontario, Canada) [19].

**Figure 3:**
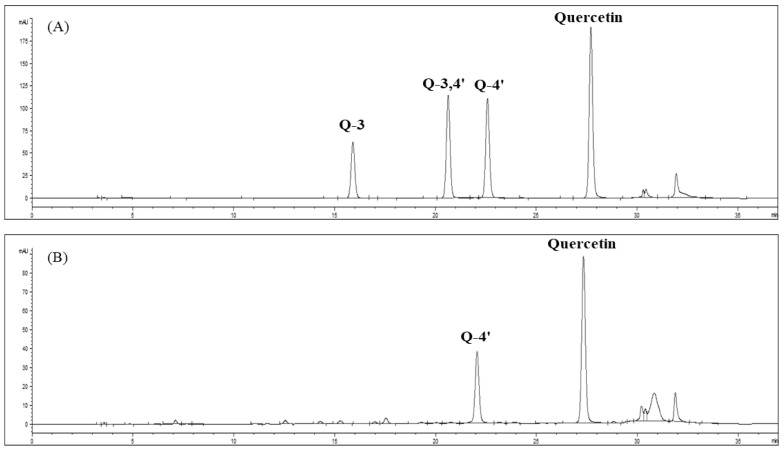
HPLC chromatograms of quercetin-3-O-glucoside, quercetin-3,4’-O-glucoside, quercetin-4′-O-glucoside, and quercetin standards (**A**) and SWE extracts from onion skin (**B**).

Quantitative analysis of quercetin levels in the onion-skin extracts was performed using HPLC (1200 series, Agilent Technologies, Santa Clara, CA, USA), a CAPCELL PAK C18 UG120 column (4.6 mm × 250 mm, 5 μm pore size; Agilent Technologies), and a UV detector (Variable Wavelength Detector, Agilent Technologies). The mobile phase consisted of (A) 5% formic acid and (B) 100% methanol. The flow rate was 0.8 mL/min, the injection volume was 10 μL, and the absorbance was measured at 360 nm. The extraction yield was calculated using standard quercetin and quercetin-4′-O-glucoside curves (Figure 3). The calibration curves were obtained using quercetin (*R*^2^ = 1) and quercetin-4′-O-glucoside (*R*^2^ = 0.999) at concentrations of 0.1, 0.5, 2, 10, 20, and 50 mg/L. The calibration curves for quercetin and quercetin-4′-O-glucoside extracted from onion skin were y = 49.685x − 1.497, and y = 30.446x − 1.322, respectively.

### 2.7. Scanning Electron Microscopy

The impact of PEF pretreatment on the physical properties of the onion skins was determined and the mechanism underlying the extraction procedure was explored with the aid of scanning electron microscopy (SEM; TM3030plus, Hitachi, Japan). The PEF-treated onion-skin samples were first allowed to dry for 24 h at room temperature, and then the non-coated samples were observed by SEM under vacuum conditions using an accelerating voltage of 5.0 kV and at magnifications of 200× and 500×.

### 2.8. Data Analysis

Each experiment was replicated more than three times. The optimum conditions (i.e., extraction temperature, extraction time, electric field strength, and duration) were chosen based on the highest quercetin contents. All data were expressed as mean ± standard-deviation values, and were analyzed using Duncan’s multiple-range test. Statistical significance was verified at *p* < 0.05. All of the statistical analyses were conducted using SPSS statistical software (IBM, Chicago, IL, USA).

## 3. Results and Discussion

### 3.1. Characterization of PEF-Induced Damage in the Cells of Onion Skins

**Figure 4:**
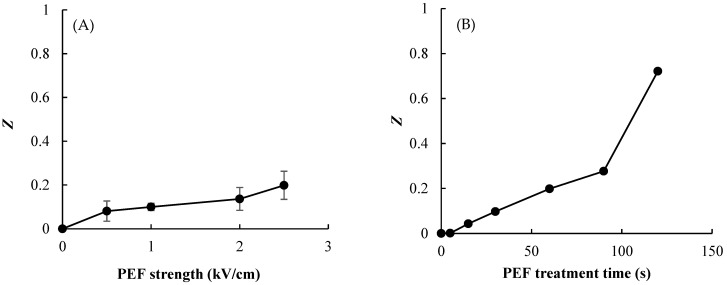
Effect of PEF strength (**A**) and treatment time at 2.5 kV/cm (**B**) on the electrical conductivity disintegration index (*Z* value) of onion skin. Data are mean and standard deviation values.

The destruction of onion-skin cells was measured using the electrical conductivity disintegration index, *Z*, which has been used previously to measure the extent of cellular destruction in various vegetable tissues [16]. Figure 4 shows the *Z* value of the onion-skin tissue according to PEF *E* value and treatment time. The results demonstrate that the physical damage to onion skin caused by PEF depends on the pulsed *E* value and PEF treatment time, with *Z* increasing with increasing pulsed *E* and processing time. The values of *Z* moved toward disintegration (i.e., increasing toward a value of 1) with the increasing PEF treatment time, and was markedly increased at the longest treatment time of 120 s. The value of *Z* was the highest (0.72) with an *E* value and treatment time of 2.5 kV/cm and 120 s, respectively (Figure 4B). This increase in *Z* values means that PEF pretreatment caused electroporation and damage such as scratches or cracks on the surface of the onion skin, enabling intracellular material leakage. The general relationships among *Z*, *E*, and treatment time observed in this study were consistent with those found previously for onions and potatoes [20,21].

### 3.2. Effect of PEF as a Pre-Treatment and SWE on Extraction of Quercetin from Onion Skin

#### 3.2.1. Effect of Pulsed *E* Value on SWE

**Figure 5:**
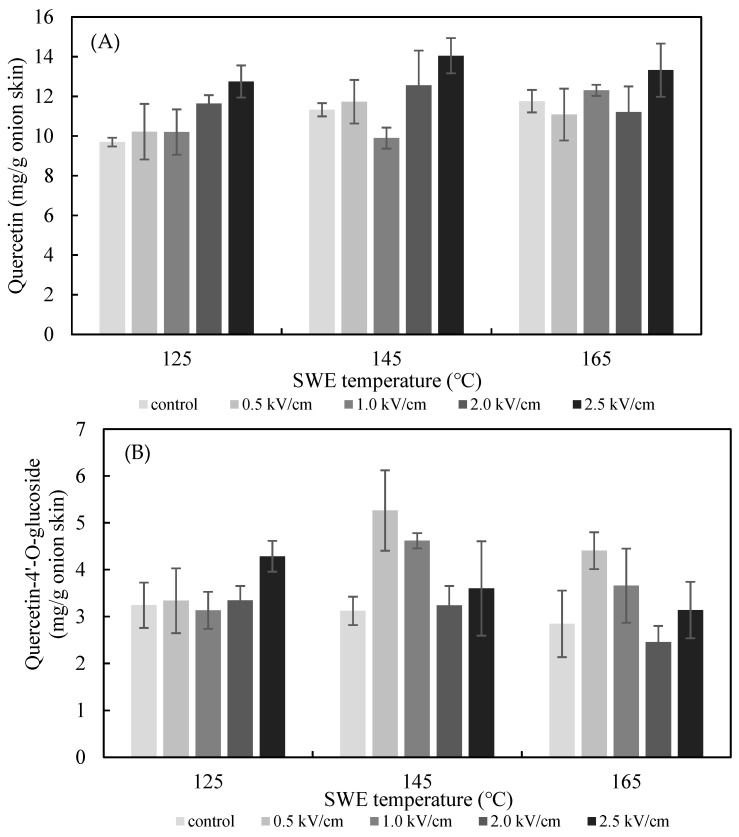

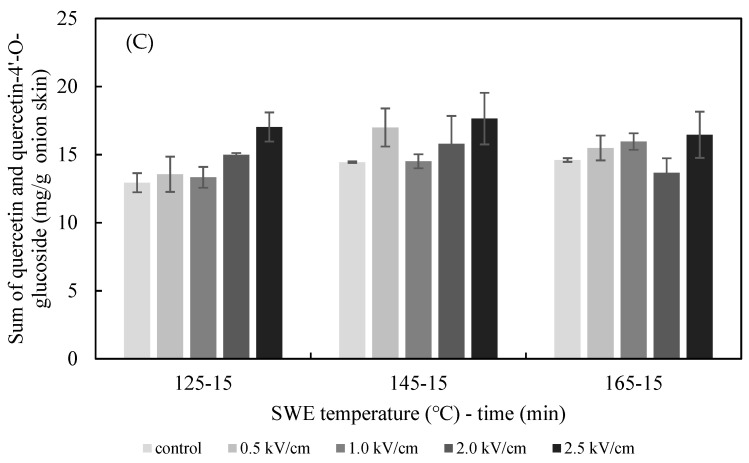
Effect of PEF strength (0.5–2.5 kV/cm) on the yield of quercetin (**A**), quercetin-4′-glucoside (**B**), and total quercetin (**C**) achieved after 15 min of SWE carried out at 125 °C, 145 °C, and 165 °C.

The effects of the PEF *E* value on the SWE yield of quercetin and quercetin-4′-glucoside are presented in Figure 5. The extraction yield of quercetin from the onion skin increased gradually as the PEF *E* increased from 0.5 to 2.5 kV/cm. The yield was significantly greater than what was achieved in the non-PEF-treated samples for all values of E. The optimum extraction conditions for quercetin from onion skin were found to be pretreatment with PEF at 2.5 kV/cm, followed by SWE at 125 °C (12.75 ± 0.81 mg/g dried onion skin), 145 °C (14.05 ± 0.89 mg/g dried onion skin), and 165 °C (13.32 ± 1.34 mg/g dried onion skin). In addition, analysis using Duncan’s test (*p* < 0.05) revealed a significantly higher content of quercetin in the extract after the onion skins were pretreated with PEF at *E* = 2.5 kV/cm compared with the controls. Therefore, the most suitable excitation value of *E* for the PEF-aided extraction of quercetin from onion skins was 2.5 kV/cm. In line with the findings of previous studies, the amount of the flavonoid extracted increased with the *E* value [16]. The extraction yield of quercetin-4′-glucoside increased gradually as the PEF *E* increased from 0.5 to 2.5 kV/cm at 125 °C. The optimum extraction condition for quercetin-4′-glucoside was found to be for pretreatment with PEF at 0.5 kV/cm, followed by SWE at 145 °C.

Previous studies have shown that mass transfer occurs more actively following the induction of membrane damage in plant tissue with the aid of a high-strength PEF. Ion leakage from onion tissue increased with the PEF strength, and a little higher electric field strength resulted in cell destruction at the same level, even though the pulse was low [22]. In addition, it was confirmed that when PEF treatment was performed on sweet potatoes, the transmembrane potential increased with *E*, resulting in increased electroporation and cell apoptosis [23]. Furthermore, the present findings also show that a high value of *E* increased the degree of membrane permeabilization. Thus, PEF treatment caused larger openings on the cell membrane and increased mass transfer so that moisture, flavor, or nutrient compounds present inside the cell could escape. The emission efficiency of ionic compounds during electrical auxiliary extraction depended mainly on both the value of *E* and the PEF treatment time [24].

#### 3.2.2. Effect of PEF Treatment Time on SWE

**Figure 6:**
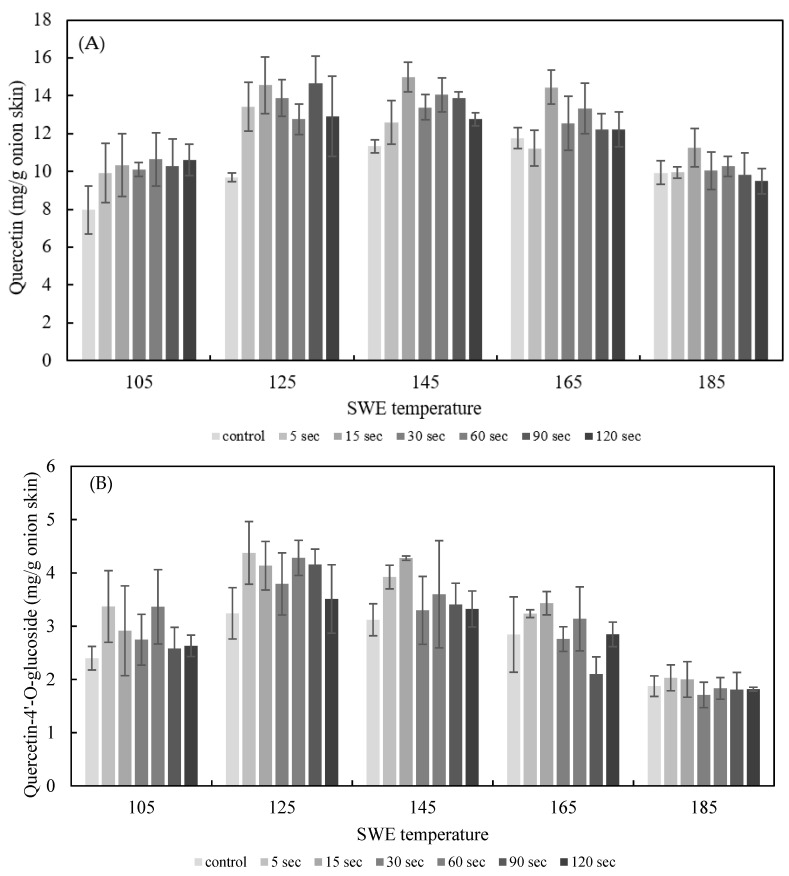

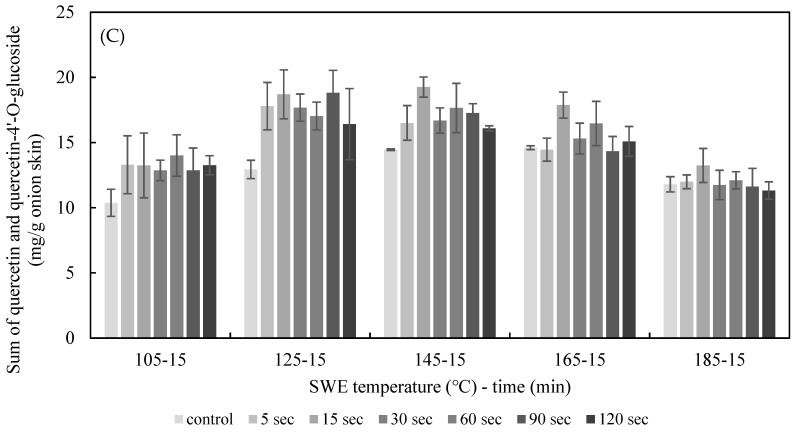
Effect of PEF treatment time on the yield of the sum of quercetin (**A**), quercetin-4′-O-glucoside **(B**), and total quercetin (**C**) for 15 min of SWE at a PEF strength of 2.5 kV/cm.

The effect of PEF treatment time on SWE was explored by applying PEF to onion skins for durations of 5 s and 120 s at *E* = 2.5 kV/cm. The quercetin and quercetin-4′-glucoside content of the onion skin extracted by SWE at varying temperatures (105–185 °C) for 15 min is shown in Figure 6. The yield of quercetin and quercetin-4’-glucoside in extracts from onion skin treated with PEF was higher than under the control conditions. The optimum conditions for quercetin extraction were PEF at 2.5 kV/cm for 15 s, and SWE at 145 °C for 15 min, which yielded 19.25 ± 0.77 mg/g dried onion skin; the optimum SWE temperature for the control was 165 °C, yielding 14.60 ± 0.14 mg/g dried onion skin. Thus, the addition of PEF lowered the temperature for the optimal extraction and increased the yield of quercetin by 33.22%. The optimum condition for quercetin-4′glucoside extraction was PEF at 2.5 kV/cm for 5 s, and SWE at 142 °C for 15 min, which yielded 4.38 ± 0.59 mg/g dried onion skin.

These findings clearly demonstrate the benefits of PEF pretreatment for SWE of quercetin and quercetin-4’-O-glucoside from onion skin. When used as a pretreatment for extraction, PEF induced the structural destruction of the cell membranes and promoted the leakage of intracellular compounds. Electrical treatment (e.g., electroplasmolysis or electropermeabilization) of fresh vegetables is an effective way of facilitating subsequent extraction by destroying cell membranes [21,25]. Cells are generally composed of cell walls surrounding a plasma membrane, contained within which is the liquid cytoplasm. Destruction of plant cell membranes by PEF can be reversible, whereby the cell membrane is resealed after treatment, or irreversible, whereby cells dissolve or rupture, depending on the function of the electrical protocol used [26].

The results of the present study support the hypothesis that PEF induces tissue damage to the onion skin. It has been reported that PEF treatment promotes the emission of intracellular compounds from cells by increasing the permeability of the cytoplasmic membrane, thus improving the extraction rates and yields of phenolic compounds [16]. The application of PEF increases mass transmission and thus increases the yield of plant-cell extracts [27]. The increased permeability of onion-skin tissue due to PEF pretreatment can help subcritical aqueous solvents more easily penetrate the onion skin and help extract the quercetin that is contained within the onion cell-wall matrix.

### 3.3. Destruction of the Onion Skin Cell Membranes by PEF

#### 3.3.1. Correlation between Quercetin Extraction Efficiency and Cell Electrical Conductivity Disintegration Index

**Figure 7:**
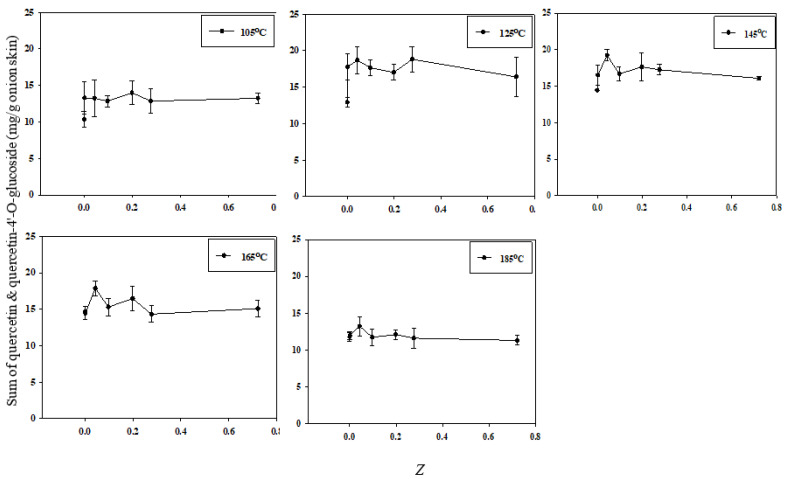
Correlation between the total quercetin extraction efficiency and cell disintegration index *Z* according to the SWE temperature.

This study used the electrical conductivity disintegration index (*Z*) to assess the integrity and disintegration of the cell membranes. The correlation between total quercetin extraction efficiency and the value of *Z* is shown in Figure 7. At all SWE temperatures except 105 °C, the extraction efficiency was the highest at *Z* = 0.043. In addition, extraction at a low temperature (125 °C) significantly increased the extraction efficiency when *Z* was increased from the very low value of 0.001429. A lower extraction temperature was associated with a higher extraction efficiency with only slight increases in *Z*; conversely, as the extraction temperature increased, the extraction efficiency was reduced. Therefore, the extraction efficiency of quercetin depends on both the value of *Z* and the temperature at which SWE is conducted.

The degree of ion leakage in PEF-treated onion tissue was significantly higher than that in the control group, and this trend was similar to that observed with the increase in *Z* [28]. PEF treatment enables a high extraction efficiency even at low temperatures by inducing cell membrane permeation to improve the dehydration and efficiency of extraction of intracellular compounds from plant materials. Together, these findings confirm that cell disintegration due to PEF treatment increases the efficiency of SWE-based quercetin extraction.

The effects of PEF on the value of *Z* have been studied previously in various plant materials. In the case of sesame cake, the value of *Z* increased as a function of PEF treatment time up to a certain critical duration. For red beet, the maximum value of *Z* increased with the PEF value of *E*, and the time taken to reach that maximum *Z* value was reduced [29]. In other studies, the effect of PEF on cell destruction was confirmed by observing the impact of *E* and PEF treatment time on the value of *Z* in raw plants such as apples [30], grapes [31], chicory [32], and red beet [29].

However, the extraction efficiency decreased slightly at high values of *Z* for certain temperatures. A strong physical force, such as PEF, was applied to release the quercetin from the surface of the matrix. An elevated extraction temperature of 185 °C of subcritical water caused a decrease in the concentration of quercetin in the organic layer [2]. This degradation of quercetin is unavoidable at very high temperatures in SWE. Therefore, defining the optimal extraction temperature for analytes is very important in SWE [11].

#### 3.3.2. Scanning Electron Microscopy

**Figure 8:**
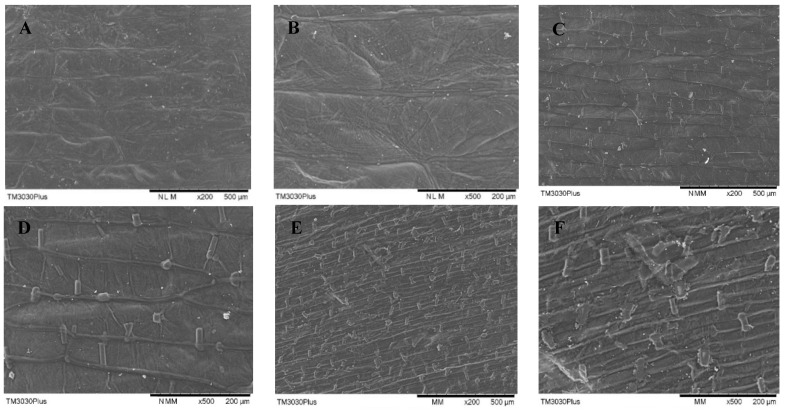
SEM micrographs of onion-skin tissue for various PEF treatment times: (**A**) control, 200×; (**B**) control, 500×; (**C**) 15 s, 200×; (**D**) 15 s, 500×; (**E**) 60 s, 200×; and (**F**) 60 s, 500×. All of the PEF samples were treated at 2.5 kV/cm.

The effects of PEF treatment time on the surface morphology of onion skin observed with the aid of SEM are shown in Figure 8. The application of PEF resulted in the disintegration of cell membranes, with the degree of fragmentation and scratching on the surface of the onion skin increasing with increasing values of *E* and treatment time. The yield of quercetin was not correlated linearly with the degree of destruction of the sample surface. Increasing the intensity of PEF treatment further increased cell-surface destruction, while the yield of quercetin decreased for treatment times longer than 15 s. These findings demonstrate that intense treatment with PEF caused quercetin leakage into the solvent. PEF as a pretreatment was a procedure to increase the mass transfer of tissues during intracellular quercetin extraction [33]. Therefore, the optimal strength of PEF treatment should be determined to increase the SWE yield of quercetin from onion skin.

The physical destruction of onion skin samples by PEF was not distinguishable with the naked eye, but was revealed upon SEM observation. PEF treatment causes disintegration of the structure of the onion-skin tissue, allowing for the quercetin contained within the cells to be readily extracted. Increased cell membrane destruction improves the penetration of the SWE solution and thus improves the extraction yields.

## 4. Conclusions

The objective of this study was to determine the effect of PEF as a pre-treatment and SWE on the extraction of quercetin and quercetin glucosides from onion skin, and to determine the optimal extraction conditions. The results of this study show that PEF treatment improved the SWE of flavonoids from onion skin, increasing the yield of total quercetin by 33.22% and lowering the temperature required for SWE from 165 °C to 145 °C compared with SWE alone. The cell electrical conductivity disintegration index data and SEM observations revealed that the main mechanism underlying the PEF-assisted extraction of quercetin from onion skin was electroporation of the cell walls, which improved the transfer of the extraction solvents into the onion-skin tissues during the SWE process. In addition, comparison of the correlation between the *Z* value and the extraction yield of the total quercetin confirmed that PEF pretreatment improved the SWE effect. These findings indicate that the combined application of PEF and SWE has the potential to efficiently extract functional compounds from plants. PEF technology is expected to be effective at preserving the important properties of food biomaterials that can decompose at high temperatures due to their low thermal stability. In addition, a different combination of ecofriendly extraction technologies such as SWE and PEF may have complementary and additive effects that will be useful for the extraction of antioxidants from plants. From a practical perspective, the development of this new processing extraction technology is critical for improving industrial-level extraction processes.

## Data Availability

No new data were created or analyzed in this study. Data sharing is not applicable to this article.

## References

[1] Munir M.T., Kheirkhah H., Baroutian S., Quek S.Y., Young B.R. (2018). Subcritical water extraction of bioactive compounds from waste onion skin. J. Clean. Prod..

[2] Kim S.W., Ko M.J., Chung M.S. (2019). Extraction of the flavonol quercetin from onion waste by combined treatment with intense pulsed light and subcritical water extraction. J. Clean. Prod..

[3] Benito-Román Ó., Blanco B., Sanz M.T., Beltrán S. (2020). Subcritical water extraction of phenolic compounds from onion skin wastes (*Allium cepa* cv. Horcal): Effect of temperature and solvent properties. Antioxidants.

[4] Waldron K. (2001). Waste utilization–useful ingredients from onion waste. Food Sci. Technol. Today.

[5] Balasundram N., Sundram K., Samman S. (2006). Phenolic compounds in plants and agri-industrial by-products: Antioxidant activity, occurrence, and potential uses. Food Chem..

[6] Williamson G., Manach C. (2005). Bioavailability and bioefficacy of polyphenols in humans. II. Review of 93 intervention studies. Am. J. Clin. Nutr..

[7] Leighton T., Ginther C., Fluss L., Harter W.K., Cansado J., Notario V. (1992). Molecular characterization of quercetin and quercetin glycosides in Allium vegetables: Their effects on malignant cell transformation. ACS Symp. Ser..

[8] Lesjak M., Beara I., Simin N., Pintać D., Majkić T., Bekvalac K., Orčić D., Mimica-Dukić N. (2018). Antioxidant and anti-inflammatory activities of quercetin and its derivatives. J. Funct. Foods.

[9] Xu D., Hu M., Wang Y., Cui Y. (2019). Antioxidant activities of quercetin and its complexes for medicinal application. Molecules.

[10] Song X., Wang Y., Gao L. (2020). Mechanism of antioxidant properties of quercetin and quercetin-DNA complex. J. Mol. Model..

[11] Essien S.O., Young B., Baroutian S. (2020). Recent advances in subcritical water and supercritical carbon dioxide extraction of bioactive compounds from plant materials. Trends Food Sci. Technol..

[12] Carr A.G., Mammucari R., Foster N.R. (2011). A review of subcritical water as a solvent and its utilisation for the processing of hydrophobic organic compounds. Chem. Eng. J..

[13] Ko M.J., Cheigh C.I., Cho S.W., Chung M.S. (2011). Subcritical water extraction of flavonol quercetin from onion skin. J. Food Eng..

[14] Lohani U.C., Muthukumarappan K. (2016). Application of the pulsed electric field to release bound phenolics in sorghum flour and apple pomace. Innov. Food Sci. Emerg. Technol..

[15] Faridnia F., Burritt D.J., Bremer P.J., Oey I. (2015). Innovative approach to determine the effect of pulsed electric fields on the microstructure of whole potato tubers: Use of cell viability, microscopic images and ionic leakage measurements. Food Res. Int..

[16] Luengo E., Álvarez I., Raso J. (2013). Improving the pressing extraction of polyphenols of orange peel by pulsed electric fields. Innov. Food Sci. Emerg. Technol..

[17] Corrales M., Toepfl S., Butz P., Knorr D., Tauscher B. (2008). Extraction of anthocyanins from grape by-products assisted by ultrasonics, high hydrostatic pressure or pulsed electric fields: A comparison. Innov. Food Sci. Emerg. Technol..

[18] Bazhal M., Lebovka N., Vorobiev E. (2003). Optimisation of pulsed electric field strength for electroplasmolysis of vegetable tissues. Biosyst. Eng..

[19] Kwak J.H., Seo J.M., Kim N.H., Arasu M.V., Kim S., Yoon M.K., Kim S.J. (2017). Variation of quercetin glycoside derivatives in three onion (*Allium cepa* L.) varieties. Saudi J. Biol. Sci..

[20] Lebovka N.I., Bazhal M.I., Vorobiev E. (2002). Estimation of characteristic damage time of food materials in pulsed-electric fields. J. Food Eng..

[21] Angersbach A., Heinz V., Knorr D. (2000). Effects of pulsed electric fields on cell membranes in real food systems. Innov. Food Sci. Emerg. Technol..

[22] Asavasanti S., Ersus S., Ristenpart W., Stroeve P., Barrett D.M. (2010). Critical electric field strengths of onion tissues treated by pulsed electric fields. J. Food Sci..

[23] Liu T., Dodds E., Leong S.Y., Eyres G.T., Burritt D.J., Oey I. (2017). Effect of pulsed electric fields on the structure and frying quality of “kumara” sweet potato tubers. Innov. Food Sci. Emerg. Technol..

[24] Vorobiev E., Lebovka N., Vorobiev E., Lebovka N. (2009). Pulsed-electric-fields-induced effects in plant tissues: Fundamental aspects and perspectives of applications. Electrotechnologies for Extraction from Food Plants and Biomaterials.

[25] McLellan M.R., Kime R.L., Lind L.R. (1991). Electroplasmolysis and other treatments to improve apple juice yield. J. Sci. Food Agric..

[26] Zimmermann U., Pilwat G., Riemann F., Zimmerman U., Dainty J. (1974). Dielectric breakdown of cell membranes. Membrane Transport in Plants.

[27] Knorr D., Angersbach A., Eshtiaghi M.N., Heinz V., Lee D.U. (2001). Processing concepts based on high intensity electric field pulses. Trends Food Sci. Technol..

[28] Asavasanti S., Ristenpart W., Stroeve P., Barrett D.M. (2011). Permeabilization of plant tissues by monopolar pulsed electric fields: Effect of frequency. J. Food Sci..

[29] Loginova K.V., Lebovka N.I., Vorobiev E. (2011). Pulsed electric field assisted aqueous extraction of colorants from red beet. J. Food Eng..

[30] Grimi N., Mamouni F., Lebovka N., Vorobiev E., Vaxelaire J. (2010). Acoustic impulse response in apple tissues treated by pulsed electric field. Biosyst. Eng..

[31] Grimi N., Lebovka N.I., Vorobiev E., Vaxelaire J. (2009). Effect of a pulsed electric field treatment on expression behavior and juice quality of chardonnay grape. Food Biophys..

[32] Zhu Z., Bals O., Grimi N., Ding L., Vorobiev E. (2015). Better damage of chicory tissue by combined electroporation and ohmic heating for solute extraction. Food Bioprod. Process..

[33] Loginova K., Vorobiev E., Bals O., Lebovka N. (2011). Pilot study of countercurrent cold and mild heat extraction of sugar from sugar beets, assisted by pulsed electric fields. J. Food Eng..

